# Fully-customized distraction assembly for maxillofacial distraction osteogenesis: a novel device and its experimental accuracy verification

**DOI:** 10.1186/s13005-020-00241-3

**Published:** 2020-11-26

**Authors:** Sang-Hoon Kang, Hye-Jin Tak, Ha-Won Park, Jin-Ung Kim, Sang-Hwy Lee

**Affiliations:** 1 Department of Oral and Maxillofacial Surgery, College of Dentistry, Yonsei University, Seoul, Republic of Korea; 2 Department of Oral and Maxillofacial Surgery, National Health Insurance Service Ilsan Hospital, Goyang, Republic of Korea; 3 Oral Science Research Center, College of Dentistry, Yonsei University, Seoul, Republic of Korea; 4 FusionTechnology Co, Ltd, #1-616, Ace Tower, Dongan-gu, Anyang, Republic of Korea; 5 DS Precision Machinery Co, Ltd - R&D Center, Shiheung City, Republic of Korea

**Keywords:** Distraction osteogenesis, Mandible, CAD/CAM, Simulation, Positioning guide, 3D printed plate

## Abstract

**Background:**

A new distraction osteogenesis assembly system comprising a fully customized CAD/CAM-based fixation unit and ready-made distraction unit was developed. The aim of this study was to introduce our new distraction system and to evaluate its accuracy level in a sampled mandibular distraction osteogenesis.

**Methods:**

Our system consists of a fully customized CAD/CAM-based fixation plate unit with two plates for each moving and anchoring part, and a ready-made distraction unit with attachment slots for fixation plates. The experimental distractions were performed on 3D-printed mandibles for one control and two experimental groups (*N* = 10 for each group). All groups had reference bars on the chin region and teeth to measure distraction accuracy. The control group had the classical ready-made distraction system, and experimental groups 1 and 2 were fitted with our new distraction assembly using a different distractor-positioning guide design. All distracted experimental mandibles were scanned by CT imaging, then superimposed on a 3D simulation to get their discrepancy levels.

**Results:**

The measured 3D distances between the reference landmarks of the surgical simulations and the experimental surgeries for the three groups were significantly different (*p* < 0.0001) by statistical analysis. The errors were greater in the control group (with a total average of 19.18 ± 3.73 mm in 3D distance between the simulated and actual reference points) than those in the two experimental groups (with an average of 3.68 ± 1.41 mm for group 1 and 3.07 ± 1.39 mm for group 2). The customized distraction assembly with 3D-printed bone plate units in group 1 and 2, however, did not show any significant differences between simulated and actual distances (*p* > 0.999).

**Conclusion:**

Our newly-developed distraction assembly system with CAD/CAM plate for the distraction osteogenesis of the mandible produced a greater level of accuracy than that of a conventional distraction device. The system appears to address existing shortcomings of conventional distraction devices, including inaccuracy in vector-controlled movement of the system. However, it also needs to be further developed to address the requirements and anatomical characteristics of specific regions.

## Background

The distraction osteogenesis in craniomaxillofacial region is used to lengthen and/or reposition the mandible, maxilla, and/or craniofacial structure for craniofacial or dentofacial deformity. It is mainly indicated for the treatment of overwhelming skeletal and/or dental discrepancy that is difficult to treat by general surgical procedure [1]. It increases skeletal stability through substantial bone formation and muscular adaptation during and after a considerable amount of surgical movement. However, difficulties in distractor application and/or directional control of distraction, which frequently result in malocclusion and/or lack of symmetry, hinder its adoption as a widespread treatment modality.

The digital diagnosis, evaluation, and simulation of craniomaxillofacial disorders using three-dimensional (3D) computed tomography (CT) and various digital technologies are coming into wide use. Designing a composite craniomaxillary-dental model by replacing the dental part of a CT model with a scanned dental model allows accurate dentofacial analysis and surgical simulation with delicate interdental occlusion. Moreover, the designed surgical device can be accurately produced following the simulation and computer-aided design (CAD) using 3D printing with stereolithography technique [2]. These digital trends can be applied to distraction as well as to the general treatment of maxillofacial deformity [3, 4].

In order to properly accomplish distraction, a surgical plan must be based on a proper preoperative diagnosis, followed by simulation to attain satisfactory esthetics and functions [5]. Surgical simulation is essential in determining the position of the osteotomy line, in estimating and performing the displacement of the distal segment in proper degree and direction, and in deciding the position of the distraction device. However, it is difficult to perform placement and directional control of the device at the operation site, partly due to the limited surgical field, especially in the case of children. The main difficulty, however, arises from the necessary customization process, i.e., bending and contouring of a factory-produced distractor with a flat plate surface to meet the curved bone surface while keeping the proposed direction of the distraction [6]. To address such problems, a computer-aided design/computer-aided manufacturing (CAD/CAM) guide can be used to position the distraction device [7–9]. We have developed such a system, which utilizes digital simulation, CAD/CAM-based guide design and 3D printing (Korean patents 10–0079973 & 0158632).

Even when the distraction device is properly positioned using a CAD/CAM guide in accordance with surgery simulation, however, the process of contouring the distractor plate to match the contour of skeletal surface and fixing it to the bone with screws give rise to difficulties in maintaining the simulated direction and position of the distractor as well as preventing possible fatigue fracture or breakdown.

We have thus further developed a new distraction osteogenesis assembly system which comprises a fully customized CAD/CAM-based fixation unit and ready-made distraction unit (PCT/KR2019/012318; Korean patent 10–2146278, 10–2146292). These can be assembled in situ after proper placement of the fixation unit with the CAD/CAM-based distractor positioning guide and screws. The system can be applied to mandible as well as other craniomaxillofacial or human body regions using the same device and following the same principles and protocol. The aim of this study was to introduce our new distraction system and to evaluate its accuracy level in a sampled mandibular distraction osteogenesis.

## Methods

### Mandibular 3D-printed models and simulation of mandibular distraction

**Fig. 1:**
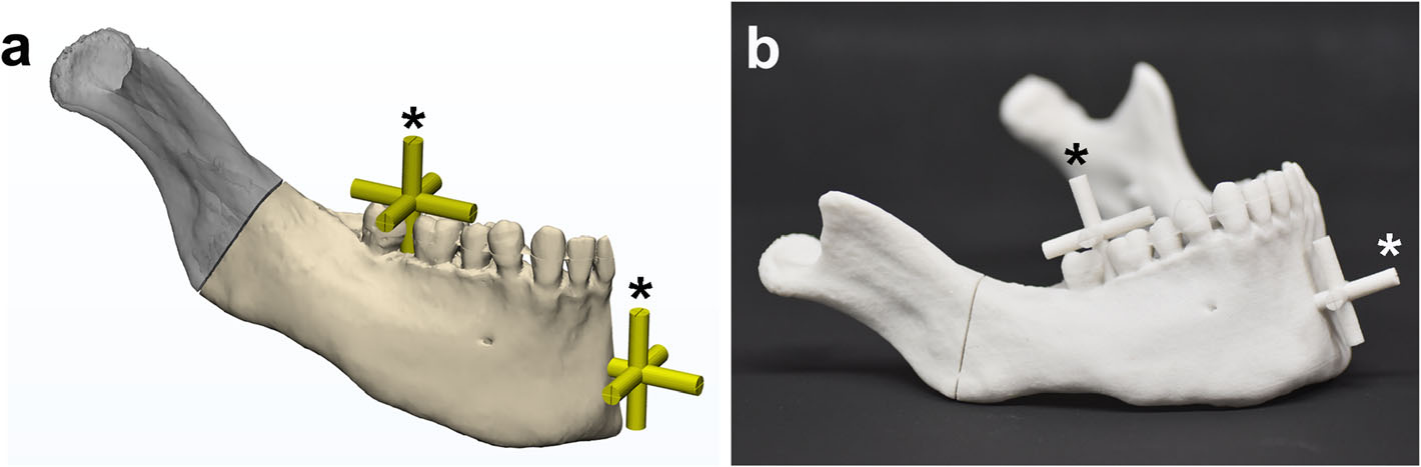
The experimental mandibular model. **a** The proximal (in gray) and distal segments (in ivory) were divided at the osteotomy line. The distal segments were designed to have two reference bars (in yellow; marked with *) in cross-bar shape to evaluate the distraction accuracy in terms of simulation-distraction distance. **b** 3D-printed mandibular model for the experiment with reference bars (*)

A mandibular 3D digital model that mimicked hemifacial microsomia was constructed from CT images with Mimics software (v.18, Materialise, Leuven, Belgium). An osteotomy plane for the distraction was designed at the mandibular angle of the model, and four cylinders were added to the osteotomy line to temporarily bridge the distal and proximal segments (Fig. 1a). The distal segments were designed to have two reference bars in a cross-bar shape with reference points at the end of each bar on the molar (representing the posterior and dental areas) and chin region (representing the anterior and skeletal areas) (Fig. 1a). Each reference bar had five points: anterior, posterior, right lateral, left lateral, and superior.

Thirty mandibular models were 3D-printed for the experiment (ProJet 360, 3D Systems, Inc., Rock Hill, SC) (Fig. 1b). Mandibular models were divided into three groups: two experimental and one control, 10 models per group, based on the design of the distractor and its positioning guide.

### Design and production of a fully-customized distraction assembly

**Fig. 2:**
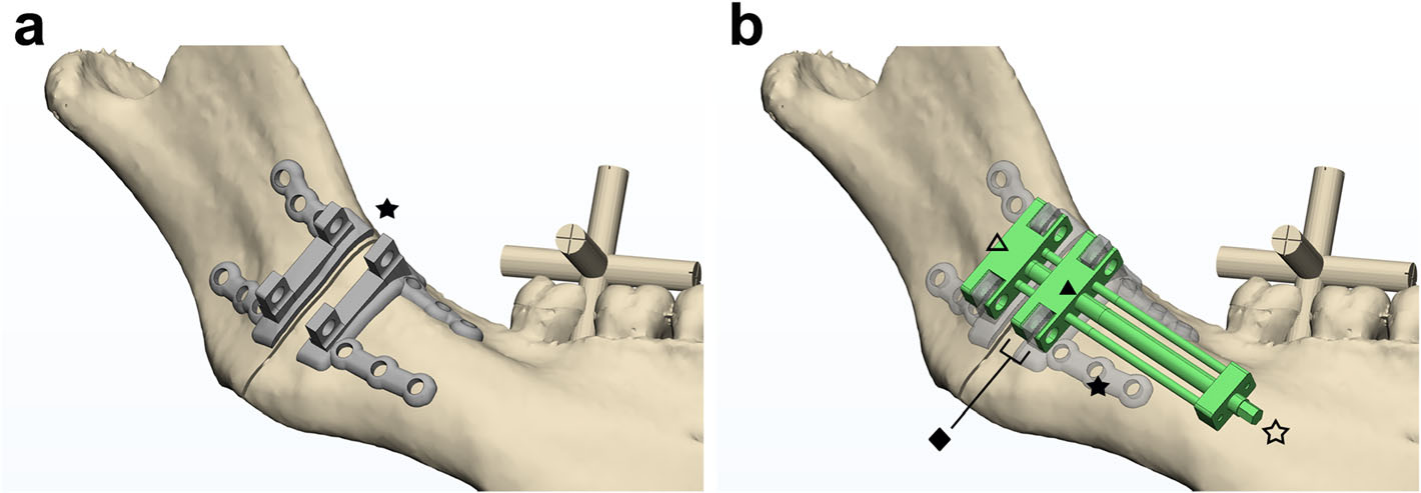
A fully customized distraction assembly. **a** A fully customized CAD/CAM-based fixation plate unit with two plates (★) for each moving and anchoring part on a three-dimensional model of the right mandibular ramus. **b** The distraction assembly system consisted of a distraction unit (☆) with attachment slots (◆) for fixation plates. A CAD/CAM-based distraction unit (green; ☆) has two slots for each moving (▲) and anchoring distractor part (△) to accept the struts of the fixation plate (★)

Our new distraction assembly system (PCT/KR2019/012318; Korean patent 10–2146278, 10–2146292) consists of a fully customized CAD/CAM-based fixation plate unit with two plates for each moving and anchoring part (Fig. 2a), and a distraction unit with attachment slots for fixation plates (Fig. 2b). Factory engineers produced the distraction unit and 3D printers did the plate: a distraction for any region of the human body can be produced using a similar unit design under the same concept. The unit has one or two slots to accept the struts of the fixation plate for each moving and anchoring part of distractor (Fig. 2b). Thus the anchor is affixed to one fixation plate and immobilized while the mobile part, assembled to another plate, rotationally drives the distal segment.

The fixation unit consisted of two plates in this study, one for the proximal or anchoring part and the other for the distal or moving part (Fig. 2a). However, various designs, such as mesh shapes, can be introduced depending on the situation. Each plate has one or two supporting struts with nearly 90 degrees of angulation to the bone surface. The plate dictates the complete contour of the bone surface at the planned plate position. This fixation plate unit can be produced by 3D titanium metal printing or other manufacturing methods including milling, and is attached to the distraction unit by screws at the slots to maintain the simulated directional cue on the bone surface.

**Fig. 3:**
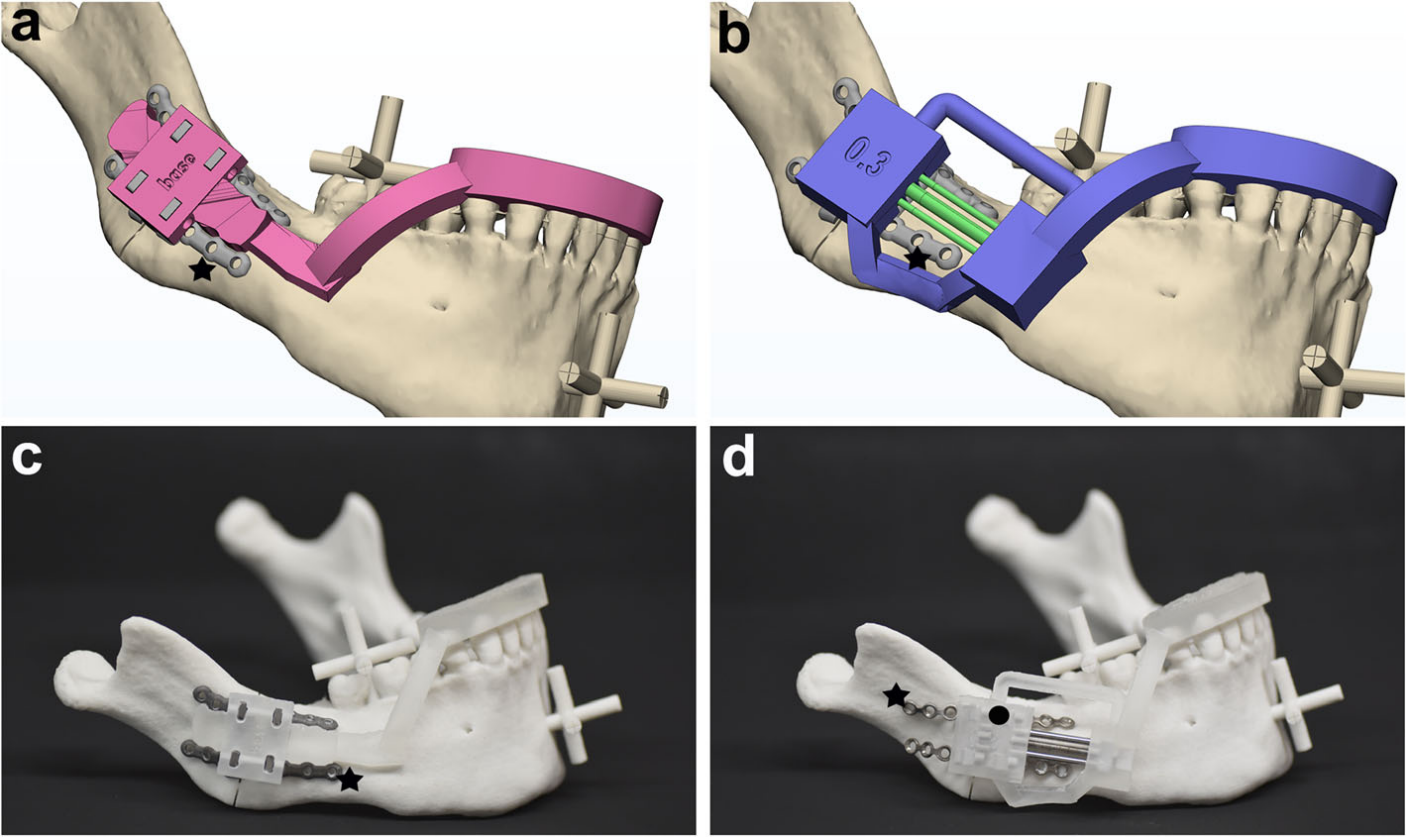
The positioning guide for distraction assembly. **a** A positioning guide (pink) designed for group 1 to position and hold the customized fixation plates (★) at the exact simulated position and direction, based on their orientation and position relative to the teeth by occlusal wafer. **b** A positioning guide (purple) with occlusal wafer designed for group 2 to position and hold both the distractor part and the customized fixation plates (★) based on the simulation scheme. **c** A 3D-printed experiment model with the customized fixation plate unit (for group 1; ★) that was guided into the planned position on the surface of the mandibular model by the in-place positioning guide (●). **d** An experiment model with the distraction unit (for control group), which was directed by the positioning guide (●) and assembled to the fixation plate (★) at the slots

Two types of guides were also designed for this study to position the distraction system in a precise simulated position and direction, relative to the teeth, through an occlusal wafer, which held the fixation plate unit only (Fig. 3a), or the whole distractor system (Fig. 3b). A 3D printer (ProJet 3500 HDMax 3D Printer, 3D Systems, Inc., Rock Hill, SC) was used to produce the designed positioning guide, which had both an occlusal wafer and a distractor holding component (Figs. 3c and d).

### Experimental group 1: distraction assembly with a positioning guide for plate unit

Two fixation plate units with four plates (i.e., two plates per fixation plate unit) were designed with 3-matic (v.18, Materialise, Leuven, Belgium) to match the contour of the mandibular bone surface, as explained previously (Fig. 2a). A 3D metal printer (SLM280, SLM Solutions Group AG, *Lübeck*, Germany) was used to produce fixation plate units in titanium, as well as the upright bar strut for holding the distractor and two bone-contoured plates (Fig. 3c).

For our experiment, the customized fixation plate unit was guided into the pre-planned surface of the 3D-printed mandibular model by the installed positioning guide device (Fig. 3c), then fixed with six titanium screws (Mini screw 8 mm, Jeil Medical, Seoul, Korea) per side. The distraction unit was affixed to the fixed plate unit with screws. The simulated mandibular osteotomy was performed by cutting the cylinders that bridged the distal and proximal part of the mandibular model at the osteotomy line.

**Fig. 4:**
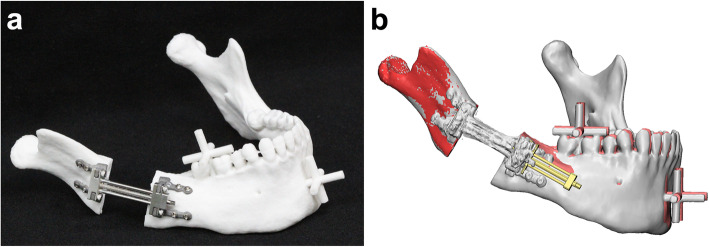
Distraction accuracy validation. **a** An experiment model is fully distracted by the activated device, driving the distal segment away from the proximal segment after a mandibular osteotomy. **b** The superimposition of mandibular models in simulation (red and coral) and post-experimental CT scanning (gray) based on the registration of their proximal parts of rami. The reference point discrepancies between the models of simulation and actual experiment were measured to evaluate distraction accuracy

**Fig. 5:**
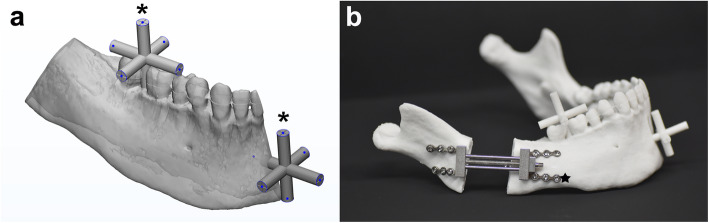
The location of refrence points and a control group sample. **a** The reference bars (*) and points (blue dots) on the distal segment of the mandibular model. The cross-shaped reference bars (*) were designed at the molar (to represent the posterior and dental area) and chin region (to represent the anterior and skeletal area). The reference points (blue dots) at the end of each bar on the simulated and experimental models were compared to measure distraction accuracy. **b** An experiment model in the control group with the conventional distractor, which was put in place by bending and contouring the fixation plate (★) to the surface of mandible with the help of a positioning guide

After the mandibular osteotomy, the distraction device was fully activated so that the mandibular distraction could be performed to drive the distal segment away from the proximal segment (Fig. 4a). The mandibular models were placed on a CT machine (Siemens Sensation 64ch MD CT scanner, Siemens AG, Erlangen, Germany) to obtain 3D CT images of the fully activated mandibular model with the distractor in place. The acquired CT images were again reconstructed to produce 3D mandibular models, and their proximal parts of rami were superimposed to those of the surgical simulation to evaluate the position of the distal parts by the point- and surface-based sequential registration in software (XOV2; Fig. 4b). In order to measure the distraction accuracy, the distal segment position was evaluated by measuring the distance between the reference points (on the reference bar) of the distal segment on a CT scan (as actual experimental data) and the corresponding points in the surgical simulation using XOV2 software (INUS Technology, Seoul, Republic of Korea) and 3-matic. Each reference bar had five points: anterior, posterior, right lateral, left lateral, and superior (Fig. 5a).

### Experimental group 2: distraction assembly with a positioning guide for both distraction and plate units

The same experiments for group 2 were performed as for group 1, except for the introduction of a positioning guide with a different design (Fig. 3b). This positioning guide could hold both the fixation and distraction unit simultaneously in their assembled state. All units were all produced using the same protocols, software, and equipment.

As the fixation plate unit was affixed to the distraction unit by screws at the slots, the positioning guide was designed using a CAD system, then 3D printed as previously described for group 1. After placement of the system with the positioning guide, the osteotomy was performed and the distraction system was fully activated. The positions of reference points on the cross-shaped reference bar of the distal segment were marked and measured as in group 1.

### Control group: conventional distraction device and positioning guide

The distractor was manufactured by the union (i.e., laser beam welding) of a normal 6-hole plate and a distraction unit designed and manufactured in the same way as that used in groups 1 and 2, except for the fixation unit assembly slots. It represented the classical distractor in terms of design and concept. The positioning guide was designed and produced in a similar manner to that for group 2, so that the distraction device could be positioned in accordance with the surgical simulation plan to match the direction of distraction and the approximate surface contour of bone at the surgical site (Fig. 3d).

By placing the distraction device on the mandibular surface with the aid of the positioning guide (Fig. 3d), one of the authors (SHK) contoured the fixation plate to fit the mandibular surface and align it in the simulated direction as much as possible. The bone plate was then fixed to the mandibular model with titanium screws as well as with a positioning guide, followed by the osteotomy and distraction, as previously described (Fig. 5b). The position of the distal segment was again evaluated by measuring the distance between the experimental data reference points on CT scan and those same points in the surgical simulation, as described for the experimental groups.

### Statistical evaluation and methods error

Statistical analyses were performed to evaluate the differences between experimental and simulated positions at the reference points (indicating distraction accuracy) for each group; the results were compared using the Kruskal–Wallis test and Dunn’s multiple comparisons test using IBM SPSS Statistics 23 (IBM Corp., Armonk, NY, USA), *p*-values < 0.05 being considered statistically significant. In addition, the error level of the experimental measurements was evaluated by measuring the distance between two reference points on the bar for both experimental and simulation sets for all three groups.

## Results

The measured 3D distance between the reference landmarks of the surgical simulation and the experimental surgery for the three groups was significantly different (*p* < 0.0001) by statistical analysis of one-way analysis of variance and Dunn’s multiple comparison (Table 1). The errors were greater in the control group (total average of 19.18 ± 3.73 mm) than those in the two experimental groups (average of 3.68 ± 1.41 mm for group 1 and 3.07 ± 1.39 mm for group 2). The customized distraction assembly with 3D-printed bone plate units in group 1 and 2, however, showed no significant differences (*p* > 0.999).

**Table 1:** Evaluation of distraction accuracy by distances between the reference points of the simulation models and those of experiments (in mm)

Reference Points^b^		Groups by distraction method^a^			Kruskal-Wallis test	Dunn’s multiple comparisons test		
Area	Location	Group 1	Group 2	Control		Group1–2	Group1-C	Group2-C
Molar	upper	2.78 ± 1.05	2.09 ± 1.19	8.64 ± 2.88	< 0.0001*	> 0.9999	0.0006*	< 0.0001*
	poserior	2.30 ± 0.67	1.99 ± 0.81	7.83 ± 2.38	< 0.0001*	> 0.9999	0.0004*	< 0.0001*
	buccal	2.43 ± 0.78	1.78 ± 1.00	9.56 ± 2.96	< 0.0001*	> 0.9999	0.0011*	< 0.0001*
	anterior	3.03 ± 1.12	2.22 ± 1.06	13.49 ± 3.28	< 0.0001*	> 0.9999	0.0007*	< 0.0001*
	lingual	2.84 ± 1.07	2.39 ± 0.92	11.91 ± 2.71	< 0.0001*	> 0.9999	0.0006*	< 0.0001*
	Subtotal	2.68 ± 0.94	2.09 ± 1.0	10.29 ± 2.84				
Chin	upper	4.44 ± 1.83	3.44 ± 1.95	24.48 ± 4.49	< 0.0001*	> 0.9999	0.0005*	< 0.0001*
	posterior	4.18 ± 1.64	3.70 ± 1.57	23.66 ± 4.30	< 0.0001*	> 0.9999	0.0003*	< 0.0001*
	right	4.39 ± 1.69	3.63 ± 2.03	25.78 ± 4.72	< 0.0001*	> 0.9999	0.0003*	< 0.0001*
	anterior	4.86 ± 2.19	4.19 ± 2.11	29.76 ± 5.08	< 0.0001*	> 0.9999	0.0002*	< 0.0001*
	left	4.62 ± 2.07	4.19 ± 1.71	27.51 ± 4.66	< 0.0001*	> 0.9999	0.0002*	< 0.0001*
	inferior	4.57 ± 2.00	4.17 ± 1.80	28.36 ± 4.88	< 0.0001*	> 0.9999	0.0001*	< 0.0001*
	Subtotal	4.51 ± 1.9	3.89 ± 1.86	26.59 ± 4.69				
Total		3.68 ± 1.41	3.07 ± 1.39	19.18 ± 3.73	< 0.0001	> 0.9999	0.0003	< 0.0001

1) *P* < 0.05*,

2) ^a^Group 1: fully-customized distraction assembly with plate-type positioning guide; Group 2: fully-customized distraction assembly with distractor-type positioning guide; Control group: conventional distractor

3) ^b^The location of reference points denote: molar upper for maxillary first molar; molar poserior for mandible second molar; molar buccal for buccal side of first molar; molar anterior for premolar area; molar lingual for lingual side of first molar; chin upper for mandibular incisor; chin posterior for mandibular symphysis; chin right for right parasymphysis;

Due to manual contouring of the fixation plate, the control group had greater errors, ranging between 7.83 mm (in the molar posterior region) and 29.76 mm (in the chin anterior region). The average error in the molar region was 10.29 mm and that at the chin was 26.59 mm, which were far greater than those of experimental groups 1 and 2 (*p* < 0.0001). The measurement points being farther from the distraction unit, the level of inaccuracy was greater in both molar and chin regions; for example, the posterior point error at the chin bar in the control group was 23.66 mm and that of the anterior point was 29.96 mm (*p* = 0.0003 for molars and 0.0288 for chin area by Mann Whitney test).

The errors were far smaller for group 1 with the positioning guide for fixation plate than for the control group; the discrepancy between the simulated and experimental positions ranged from 2.30 to 3.03 mm (with a mean of 2.68 mm) in the molar region and from 4.18 to 4.86 mm (with the mean of 4.51 mm) in the chin region. The error levels of group 2 (with positioning guide for both fixation and distraction unit) were similar to those of group 1, being 1.78–2.39 mm (in the molar region) and 3.44–4.19 mm (in the chin region). Both groups also showed greater discrepancies at those reference points farther from the distraction unit, as seen in the control group, with no remarkable difference between upper and lower or between the medial and lateral regions.

Errors during the bar length measurement of simulation and experimental data were not significantly different; the average measured distance between reference points on the bar was 20.54 mm for the simulation and 20.52 mm for the actual experimental model. The average measurement difference between the simulation and experiment reached 0.018 ± 0.763 (mean ± standard error of mean; *p* < 0.0001 by Kruskal-Wallis test).

## Discussion

The goal of this study was to introduce our newly-developed distraction assembly system and to demonstrate its applicability by evaluating its accuracy level in a sampled mandibular distraction osteogenesis. We conducted independent experimental distractions by the classical and new distraction systems, assisted by the introduction of a wafer-type positioning guide, on 3D-printed mandibular models. The newly-developed assembly system yielded significantly smaller discrepancies compared with those of conventional distraction.

The success of distraction basically depends on the biological condition of the tissues, including the skeletal and soft tissue as well as their blood supply [10]. Successful distraction is also related to the quantity and quality of bone formation and remodeling, which are associated with the duration of latency and consolidation, as well as distraction speed [11]. Finally, the stability and vector control of the distractor is critically important for the success of distraction, especially considering that the craniofacial distraction is closely related to occlusion or other functional/esthetic factors [12, 13].

Our distraction accommodates the same screw-driven linear-moving mechanical system as that of a classical distraction system. The distraction parameters of the classical system are thus suitable for use here, including the 1 mm/day distraction speed and a minimum three-month consolidation period. Moreover, our system was validated for mechanical accuracy and stability, but not for biological consistency in new bone formation. Although a similar biological response is anticipated, further animal experiments should provide more insight regarding parameters for successful distraction. Among these three major factors related to successful distraction, we focused more on the third factor, stability and vector control, since we believe the other two factors have been addressed relatively well [6, 14]. In order to address the issues of direction and stability, we previously introduced a way to transfer 3D positional information from simulation to the operation field with the aid of a positioning guide (Korean patents 10–0079973 & 0158632). Previous studies have introduced 3D-printed positioning guides [4, 7] or navigation equipment [15]. Our current study attempted two kinds of positioning guide for bone plate (in group 1) or for bone plate plus distraction device (in group 2). There were no significant differences between these two groups, suggesting that the guides functioned similarly in terms of distraction accuracy.

Appropriate materials and production methods are important issues in the development of CAD/CAM-based distraction. Medical titanium alloy or stainless steel are primary choices for producing fixation plates by 3D printing or milling. In either case, the production protocol must take into account factors including time, cost, mechanical strength, biological response, and distraction method.

In addition, positioning guides are generally 3D printed using medical resin or metal [16, 17]. Appropriate material choice depends on production requirements, biomechanical characteristics, and application, whether osteotomy guide, distraction device positioner, or drill guide for fixation plate. The guide can also be designed to accommodate positional information regarding teeth and occlusion or bone surface contour. The introduction of positioning guides for distraction can shorten the operation time and enhance accuracy. Various types of guides by CAD/CAM design will be developed [7].

Here we evaluated our new system to enhance the positional accuracy and stability of the transported distal segment, consisting of a fully-customized fixation unit, detachable distraction unit, as well as a positioning guide. One must consider existing devices to understand the advantages of this system. Conventional factory-made bone plates need to be contoured in accordance with the regional surface curvature of bone, which is inevitably limited by the greater gap between the plate and bone surface, lack of contouring proficiency and narrow operation environment. These factors frequently distort the planned position and direction of the distractor, and occasionally induce fatigue fracture at the junction between the plate and distractor during contouring or distraction [18]. We therefore produced a customized bone plate system using CAD/CAM design and 3D production by metal printing or milling, which do not require a bending process for fitting to the patient’s bone surface.

Although some shortcomings of traditional distraction devices were addressed by our previous trial with a customized guide system, the distraction device still posed difficulties in terms of customized production of a screw-based system with a mobile distal segment. To resolve this issue, we developed plate unit struts that protrude from the base of the plate to provide stable binding with the slots of the distraction unit, fixed by screws. This study also attempted to confirm solidity of assembly, but further study is needed to evaluate biomechanical tolerance and possible side effects relevant to clinical practice or surgical outcomes.

This study confirmed our system’s higher accuracy, likely due to the designed simulation scheme and accurate transport of the distracted segment (with a total mean error of 3.07 or 3.68 mm at the reference points), relative to that of a conventional distraction system (with an error of 19.18 mm; *p* < 0.0001). A study by Chen, K., et al. [8] compared the distraction accuracy of simulation and actual surgeries for seven consecutive temporomandibular joint ankyloses. Employing surgery simulation and a 3D-printed template guide, they found discrepancies in 3D positions of three reference points, i.e., menton, and right and left gonion, ranged from 0.6 to 1.9 mm. The level of accuracy for their in-vivo trial was smaller than for our in-vitro trial, but these may not be directly comparable because the measurements were made at different locations using different methods. In addition, their results mainly related to the vertical dimension due to the condylar lengthening, while ours were related to the horizontal dimension, being based on angular lengthening. The vertical dimension-related discrepancies of our study reached 3.23 (group 2), 3.93 (group 1), and 20.5 mm (control group) at the superior and inferior points. An additional reason for the discrepancy may be related to the location of the reference points and the distraction length-direction, since we performed mainly horizontal distraction with an average distraction length of 27.5 mm, whereas their vertical distraction averaged 14.9 mm.

This may be evidenced by another template-based in-vivo distraction study for condylar lengthening for hemifacial microsomia, which showed a dimension-dependent discrepancy of 0.93 mm between simulation and surgical results in the anteroposterior direction, and 4.64 mm in the horizontal plane [16]. Another study evaluated the position error of pediatric navigational distraction, finding a mean angular error of 3.7° in the horizontal plane and 6.2° in the sagittal plane, yielding 3.98 mm in distance at the distractor [5]. A study of mandibular cadaver reported the angular difference between the preoperative and postoperative vector to be + 3° for the stereolithographic guide and − 6° for the navigation procedure [19]. We believe our results are similar to or better than theirs in terms of discrepancy size. We will further develop our system through animal experiments to enhance clinical applicability as well as to prevent possible error.

The main goal of our new development was to enhance distraction accuracy by precise application and vector control, as described previously. This improved accuracy does not always guarantee the recovery of geometric morphology of the biological structure, especially in mandible. The design of our system follows the general structural pattern of a linear distraction system, which inevitably limits the ability to restore the original geometry of the biological structure. Further work will be devoted to modifying our system to accept the regional anatomical and geometrical characteristics of the skeletal structure.

## Conclusions

This study introduced a distraction assembly system with customized bone plate to address the shortcomings of conventional distraction devices, such as possible fatigue fracture, time and accuracy in contouring, firm fixation and accurate vector-controlled movement of the system. We performed experimental distractions to show significantly smaller discrepancies in the newly-developed assembly system as compared with those of conventional distraction. The new distraction system may need to be modified depending on regional characteristics of maxillary or other anatomical regions. Further consideration may be given to the temporomandibular joint, periosteum, muscle and soft tissues, and developmental validation. Finally, the development of various surgical positioning guides for the distraction procedure will allow the introduction of supplementary navigation.

## Data Availability

The datasets used and/or analyzed during the current study are available from the corresponding author upon reasonable request.
